# Serum Ferritin Levels as a Prognostic Marker for Predicting Outcomes in Children With Severe Sepsis and Their Correlation With Pediatric Sequential Organ Failure Assessment Score

**DOI:** 10.7759/cureus.84436

**Published:** 2025-05-19

**Authors:** Mansi Lal, Manjusha Goel, Neha Shrivastava, Pankaj K Pal, Monali Datta

**Affiliations:** 1 Pediatrics, Gandhi Medical College, Bhopal, IND

**Keywords:** hyperferritinemia, mortality, predictor of mortality, psofa score, sepsis, serum ferritin, severe sepsis, severe sepsis outcome, severe sepsis prognostication, survival in severe sepsis

## Abstract

Background: Severe sepsis is associated with increased mortality in the pediatric population. Potential biomarkers, such as serum ferritin, may be helpful in the timely prognostication of severe sepsis. This study aimed to evaluate the prognostic ability of serum ferritin levels in children with severe sepsis. Additionally, the study also aimed to find the correlation between serum ferritin levels and the Pediatric Sequential Organ Failure Assessment (pSOFA) score in children with severe sepsis.

Methodology: This study was conducted as an observational cross-sectional study on children admitted with severe sepsis to the pediatric intensive care unit (PICU) of a tertiary care center in Bhopal, India, over a period of 12 months. A total of 82 children aged between 1 month and 12 years who presented with severe sepsis were enrolled. Sepsis and severe sepsis were defined according to the 2005 International Pediatric Sepsis Definition Consensus criteria. The sociodemographic and clinical details of the study population were documented. Serum ferritin levels were measured within 24 hours of admission, and the pSOFA score was assessed twice, at 24 hours and 48 hours after admission, and the mean value was calculated. The final outcome was recorded in terms of survival and non-survival, and the results were compared between these two groups. Statistical analysis involved Pearson’s correlation, receiver operating characteristic (ROC) curves, and logistic regression analysis.

Results: The mean serum ferritin levels (849.55 ± 300.05 ng/mL vs. 398.45 ± 97.53 ng/mL) and pSOFA scores (13.50 ± 0.53 vs. 7.33 ± 2.31) were found to be significantly higher among non-survivors compared to survivors (p < 0.05). ROC curve analysis revealed good predictive ability of serum ferritin levels for mortality in children with severe sepsis. At a cut-off of >504 ng/mL, serum ferritin showed an area under the curve (AUC) of 0.992, with sensitivity of 100% and specificity of 84.3%. A significant correlation was found between serum ferritin levels and pSOFA score (r = 0.70 to 0.90; p < 0.05). At a cut-off of 12, the pSOFA score showed significant predictive ability for hyperferritinemia (>504 ng/mL), with an AUC of 0.901, sensitivity of 79.5%, and specificity of 100%. Logistic regression analysis documented serum ferritin levels and pSOFA score as independent markers of mortality. The risk of mortality was 8.3 times higher in patients with a mean pSOFA score >12. With respect to serum ferritin level, hyperferritinemia (>504 ng/mL) was associated with a 1.843 times higher risk of mortality.

Conclusion: High serum ferritin levels (>504 ng/mL) and pSOFA score >12 are both excellent and independent predictors of mortality in children with severe sepsis. A pSOFA score >12 has significant correlation with hyperferritinemia (serum ferritin >504 ng/mL). Incorporating these measurements in the care of children with severe sepsis may enhance risk stratification and support early intervention.

## Introduction

Sepsis is a life-threatening medical condition that occurs when the body’s dysregulated response to an infection causes widespread inflammation. Therefore, sepsis in the pediatric population is a major public health problem, associated with significant mortality and morbidity. Literature suggests that severe sepsis accounts for more than 8% of admissions to the pediatric intensive care unit (PICU) and is responsible for more than 4.5 million deaths globally in the pediatric age group [1-3]. The mortality due to sepsis can be as low as 4% or as high as 50%, depending on the severity of illness, level of care, and associated risk factors [4].

Early diagnosis and timely prognostication of sepsis severity are essential for reducing the morbidity and mortality associated with it. This is where the role of sepsis biomarkers becomes essential for prognostication. These biomarkers allow early identification of severe sepsis, which can guide timely intensification of medical intervention to improve outcomes and survival.

Recently, serum ferritin has emerged as a potential biomarker of disease severity as well as a prognostic marker for predicting outcomes in critically ill children [5]. Serum ferritin is normally present in the body and reflects iron stores. However, in sepsis, pro-inflammatory cytokines trigger the synthesis and release of ferritin. Thus, ferritin acts as an acute-phase protein in sepsis [6].

The pSOFA (Pediatric Sequential Organ Failure Assessment) score is a tool used to assess the severity of organ dysfunction and predict prognosis in pediatric patients. In critically ill children, organ dysfunction assessment helps in predicting outcomes, guiding resuscitation timing, and improving quality of care [7]. Clinical correlation with sepsis biomarkers can further enhance the prognostic ability in managing children with severe sepsis.

Thus, this study was conducted at a tertiary care center to evaluate the prognostic ability of serum ferritin when estimated within five days of illness onset in children aged one month to 12 years with severe sepsis admitted to the PICU. The study also aimed to find a correlation between serum ferritin levels and the pSOFA score in children with severe sepsis.

## Materials and methods

This study was conducted as an observational cross-sectional study on patients admitted with severe sepsis to the PICU, Department of Pediatrics, at a tertiary care center. The study was carried out over a 12-month period from May 2023 to May 2024. The study population consisted of 82 children admitted to the PICU with severe sepsis. Sepsis and severe sepsis were defined according to the 2005 International Pediatric Sepsis Definition Consensus criteria (Table 1) [1].

**Table 1:** International Pediatric Sepsis Definition Consensus criteria SIRS: systemic inflammatory response syndrome, WBC: white blood cell, SIRS: systemic inflammatory response syndrome, MODS: multiple organ dysfunction syndrome.

Term	Definition
Systemic inflammatory response syndrome (SIRS)	Defined as the presence of two or more clinical criteria, one of which must be either an abnormal temperature or white blood cell (WBC) count. These include pyrexia (>38.5 °C) or hypothermia (<36 °C), age-dependent tachycardia or bradycardia, tachypnea or the need for mechanical ventilation, and an abnormal WBC count or more than 10% immature neutrophils.
Sepsis	SIRS and suspected or confirmed infection
Severe sepsis	Sepsis plus one of the following: cardiovascular organ dysfunction or respiratory dysfunction, or 2 or more non-cardiorespiratory organ system dysfunction
Septic shock	Sepsis and cardiovascular dysfunction, defined as either hypotension, receipt of vasoactive medication, or impaired perfusion despite fluid resuscitation
Multiple organ dysfunction syndrome (MODS)	A clinical syndrome characterized by the development of progressive and potentially reversible physiologic dysfunction in 2 or more organ systems that is induced by a variety of acute insults.

The sample size was estimated using the following formula: 

$n=\frac{z^{2}pq}{d^{2}}$

where n is the sample size, z = 1.96 at 95% confidence interval, p is the predetermined value of sensitivity = 74% [8], q = 1 − p = 26%, and d is the allowable error = 10%.

Based on the above formula, the sample size was estimated to be 74, and an additional 10% was included to account for potential loss to follow-up. Thus, the final sample size was 82.

All children aged between 1 month and 12 years, admitted to the PICU and presenting with severe sepsis within five days of illness onset, were included. Children with chronic organ dysfunction (such as chronic liver, kidney, lung, or heart disease), those who received blood transfusion in the last four months, those with inherited or acquired disorders of iron metabolism, diagnosed or suspected malignancy, autoimmune disease, or PICU stay less than 24 hours were excluded.

After obtaining ethical clearance from the institute’s ethical committee, all patients fulfilling the inclusion criteria were enrolled, and written informed consent was obtained from the parents or guardians. Using a pretested proforma, sociodemographic details were documented. All patients underwent detailed general and systemic examination, and findings were recorded. Routine blood investigations and serum ferritin levels were measured within 24 hours of admission. To predict prognosis, the pSOFA score was used. It was assessed twice, at 24 hours and at 48 hours, and the mean score was calculated. All patients were followed up during their hospital stay, and final outcomes were documented.

Data were compiled using MS Excel (Microsoft Corporation, Redmond, Washington) and analyzed using IBM SPSS Statistics for Windows, Version 20 (Released 2011; IBM Corp., Armonk, New York). Categorical data were expressed as frequency and proportion, whereas continuous data were expressed as mean and standard deviation. Data were analyzed using the chi-square test for comparing categorical variables, and the independent t-test was used for continuous variables. Receiver operating characteristic (ROC) curve analysis was performed to determine the predictive capacity of serum ferritin and pSOFA score for mortality. ROC curve analysis was also used to assess the predictive ability of the pSOFA score in determining hyperferritinemia. Pearson’s correlation was employed to evaluate the relationship between pSOFA score and serum ferritin levels. The area under the curve (AUC) was calculated with a 95% confidence interval (CI). Sensitivity, specificity, and optimal cutoff points were calculated using the Youden Index. Logistic regression analysis was performed to determine whether serum ferritin levels and pSOFA score were independent markers of mortality. A P-value <0.05 was considered statistically significant.

## Results

The study was conducted on a total of 82 pediatric patients admitted to the PICU with severe sepsis after fulfilling the inclusion criteria. The mean ± SD age of patients with severe sepsis was 2.87 ± 1.4 years, and 49 (59.76%) cases were males, with a male-to-female ratio of 1.5:1. While pneumonia and multiple organ dysfunction syndrome (MODS) were more common among males, the incidence of urinary tract infection (UTI) was higher in females. These findings were statistically significant (P < 0.05).

The mean ± SD serum ferritin level was 568.99 ± 296.06 ng/mL, with no significant difference between males and females. The mean ± SD pSOFA score was 9.665 ± 3.53, and males had significantly higher values compared to females (P < 0.05). Mortality was reported in 31 (37.8%) cases, with no significant difference between males and females (Table 2).

**Table 2:** Demographic and clinical profile of study subjects Statistical tests used were the chi-square test for categorical variables and an independent t-test for continuous variables. P-value <0.05 is considered significant. MODS: multiple organ dysfunction syndrome, pSOFA: Pediatric Sequential Organ Failure Assessment score.

Variables		Males (n=49)	Females (n=33)	Total (n=82)	P-value
Age (years)	1 month to 1 year	15 (30.6%)	11 (33.3%)	26 (31.7%)	0.84
	1 to <5 years	24 (49%)	14 (42.4%)	38 (46.3%)	
	5 to 12 years	10 (20.4%)	8 (24.2%)	18 (22%)	
Clinical presentation	Severe sepsis	49 (100%)	33 (100%)	82 (100%)	
	Septic shock	29 (59.2%)	15 (45.5%)	44 (53.7%)	0.22
	MODS	21 (42.9%)	6 (18.2%)	27 (32.9%)	0.02
Primary system infected	Pneumonia	34 (69.4%)	11 (33.3%)	45 (54.8%)	0.001
	Meningitis	13 (26.5%)	17 (51.5%)	30 (36.6%)	0.09
	Intra-abdominal infections	2 (4.1%)	1 (3%)	3 (3.7%)	0.83
	Urinary tract infection	0 (0%)	4 (12.1%)	4 (4.9%)	0.01
Serum ferritin (ng/mL)	Mean ± SD	596.04±263.55	528.82±338.96	568.99±296.06	0.32
pSOFA score	At 24 hours	10.14±2.54	8.67±2.78	9.55±2.72	0.015
	At 48 hours	10.45±4.35	8.73±4.47	9.76±4.44	0.085
	Mean	10.296±3.36	8.727±3.61	9.665±3.53	0.048
Outcome	Survivors	28 (57.1%)	23 (69.7%)	51 (62.2%)	0.25
	Non-survivors	21 (42.9%)	10 (30.3%)	31 (37.8%)	

The mean ± SD serum ferritin levels and mean ± SD serial pSOFA scores at 24 and 48 hours were found to be significantly higher among non-survivors compared to survivors (P < 0.05) (Table 3).

**Table 3:** Comparison of serum ferritin and pSOFA score by outcome (survivors vs. non-survivors) Statistical test used: independent T-test. P-value <0.05 is considered significant. pSOFA: Pediatric Sequential Organ Failure Assessment score.

S. no.	Variable	Category	Survivors (n=51)	Non-survivors (n=31)	T-test	P-value
1	Serum ferritin (ng/mL)	-	398.45±97.53	849.55±300.05	9.94	0.001
2	pSOFA score	At 24 hours	8.04±2.37	12.03±0.61	9.17	0.001
		At 48 hours	6.63±2.28	14.90±0.79	19.47	0.001
		Mean	7.33±2.31	13.50±0.53	14.58	0.001

**Figure 1:**
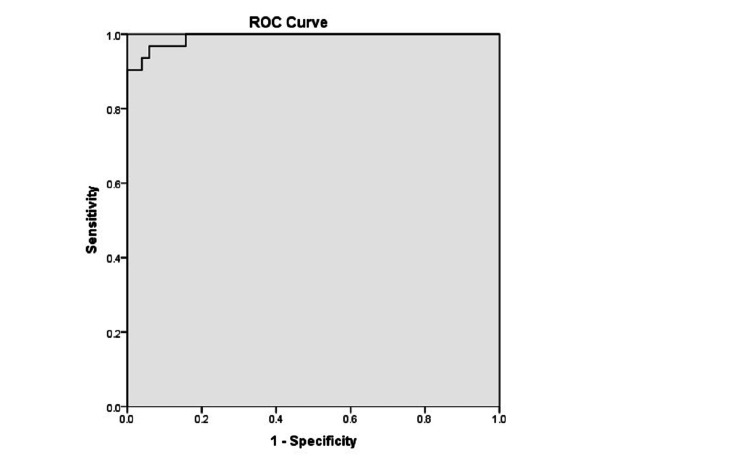
ROC curve for serum ferritin in predicting mortality in children with severe sepsis The area under the curve (AUC = 0.992) was calculated with a 95% confidence interval (CI). ROC curve: receiver operating characteristic curve.

**Figure 2:**
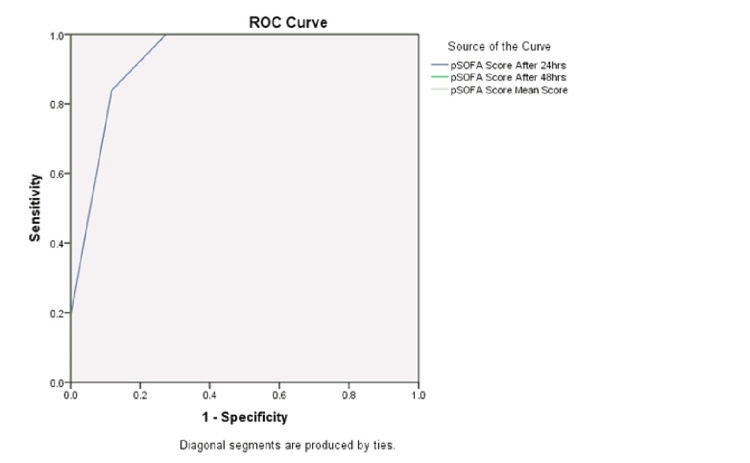
ROC curve for pSOFA in predicting mortality in children with severe sepsis The area under the curve (AUC) is 0.93 at 24 hours and 1 for both mean and at 48 hours. AUC was calculated with a 95% confidence interval (CI). pSOFA: Pediatric Sequential Organ Failure Assessment score, ROC curve: receiver operating characteristic curve.

The ROC curve analysis of serum ferritin levels for predicting mortality identified significant predictive ability at >504 ng/mL as the optimal cutoff, with a Youden Index of 0.83 and an AUC of 0.992 (95% CI: 0.979-1) (Table 4, Figure 1). At this threshold, the sensitivity was 100% and the specificity was 84.3%, indicating that serum ferritin levels >504 ng/mL are strongly associated with a higher risk of mortality. Similarly, for the pSOFA score, a value of 12 was identified as the optimal cutoff, with a Youden Index of 0.72 -1 and AUC of 0.9-1 (95% CI 0.878-1). This threshold demonstrated 100% sensitivity and specificity in predicting mortality with a pSOFA score >12 at 48 hours and in the mean value (Table 4, Figure 2).

**Table 4:** ROC curve analysis for predicting the role of serum ferritin and pSOFA for mortality The area under the curve (AUC) was calculated with a 95% confidence interval (CI). P-value <0.05 is considered significant. pSOFA: Pediatric Sequential Organ Failure Assessment score, ROC curve: receiver operating characteristic curve.

Variable		Area	Std. Error	P value	95% CI	Cutoff	Sensitivity %	Specificity %	PPV %	NPV %
Serum ferritin		0.992	0.007	0.0001	0.979-1.0	504.00	100	84.3	79.5	100
pSOFA	At 24 hours	0.93	0.027	0.0001	0.878-0.983	12.00	83.9	88.2	88.5	80.6
	At 48 hours	1.0	0.00	0.0001	1.0-1.0	12.00	100	100	100	100
	Mean	1.0	0.00	0.0001	1.0-1.0	12.00	100	100	100	100

The logistic regression analysis revealed that hyperferritinemia (serum ferritin >504 ng/mL) was associated with a 1.843 times higher risk of mortality (P<0.05). Both Serum ferritin (>504 ng/mL) and pSOFA score (>12) were independent predictors of mortality in children with severe sepsis (Table 5).

**Table 5:** Logistic regression analysis of serum ferritin and pSOFA to determine the probability of mortality Statistical test applied: Logistic regression analysis. Odds Ratio (OR) calculated with a 95% confidence interval (CI).p value <0.05 is considered significant. pSOFA: Pediatric Sequential Organ Failure Assessment score

Outcome (mortality)			OR	95% CI	P-value
pSOFA	At 24 hours	>12	1.94	1.03-2.82	0.001
	At 48 hours	>12	6.3	6.1-7.15	0.001
	mean	>12	8.3	7.4-9.1	0.001
Serum ferritin		>504	1.843	1.7-1.9	0.001

With the Pearson correlation coefficient (r) of >0.7, there is a high positive correlation of serum ferritin levels with pSOFA score at 48 hours, as well as mean pSOFA score (P<0.05). Meanwhile, with an r-value of 0.652, there is a moderate positive correlation between pSOFA at 24 hours and serum ferritin levels (P<0.05) (Table 6). 

**Table 6:** Correlation between serum ferritin levels and pSOFA score Statistical test applied: Pearson’s correlation test. P-value <0.05 is considered significant. pSOFA: Pediatric Sequential Organ Failure Assessment score.

pSOFA	r	r^2^	Adjusted r^2^	Standard error of the estimate	P-value
At 24 hours	0.652	0.425	0.418	2.077	0.0001
At 48 hours	0.757	0.572	0.567	2.924	0.0001
Mean	0.731	0.535	0.529	2.422	0.0001

The logistic regression analysis revealed that a pSOFA score of >12 was an independent predictor of hyperferritinemia (serum ferritin >504 ng/mL). A mean pSOFA score of >12 is associated with a 7.95 times higher risk of hyperferritinemia (P<0.05) (Table 7).

**Table 7:** Logistic regression analysis of pSOFA to determine the probability of hyperferritinemia. Statistical test applied: logistic regression analysis. Odds ratio (OR) calculated with a 95% confidence interval (CI). P-value <0.05 is considered significant. pSOFA: Pediatric Sequential Organ Failure Assessment score.

Hyperferritinemia (Serum ferritin >504 ng/mL)		OR	95% CI	P-value
pSOFA at 24 hours	>12	3.762	1.54 to 8.78	0.007
pSOFA at 48 hours	>12	7.56	6.5-8.55	0.001
Mean pSOFA	>12	7.95	6.7-9.2	0.001

## Discussion

Our study aimed to evaluate the prognostic ability of serum ferritin in children with severe sepsis, along with analyzing the correlation between serum ferritin levels and the pSOFA score. 

Our findings revealed significantly higher levels of serum ferritin in severe sepsis patients with adverse outcomes. The mean serum ferritin levels among non-survivors were 849.55 ± 300.05 ng/mL compared to 398.45 ± 97.53 ng/mL among survivors (P < 0.05). Using ROC curve analysis, our study found a significant predictive ability of serum ferritin levels for mortality in children with severe sepsis. The cut-off level for predicting mortality was identified using the Youden Index as 504 ng/mL (AUC: 0.992, sensitivity 100%, specificity 84.3%).

A study by Kulkarni et al. [9] also documented serum ferritin levels above 300 ng/mL to be significantly associated with higher mortality rates. i.e., about 64.3% of non-survivors had serum ferritin levels above 300 ng/mL compared to 35.7% of survivors (P < 0.05). The findings of the present study were concordant with the study by Sarkar et al. [10], in which mean serum ferritin levels were significantly higher among non-survivors (7553 ± 2949 ng/mL) compared to survivors (1293 ± 79 ng/mL; p < 0.05). In their study, ROC curve analysis revealed excellent predictive ability of serum ferritin for mortality (AUC: 0.974; 95% CI: 0.952-0.996; p < 0.05). The best cut-off was much higher compared to our study (2375 ng/mL), with a sensitivity of 96.7% and specificity of 88%.

The findings of our study were also supported by Tonial et al. [11], where serum ferritin levels among survivors were 138 ng/mL compared to 586 ng/mL in non-survivors (P < 0.01). Serum ferritin levels within 48 hours of admission were identified as a significant predictor of mortality (OR: 5.170), and a tenfold increase in serum ferritin levels was associated with a fivefold increase in death risk. ROC curve analysis revealed serum ferritin to be a good predictor of mortality (AUC: 0.787; 95% CI: 0.737-0.830; p < 0.0001). A study by Garcia et al. [12] also showed a significant association between higher serum ferritin levels and mortality. The mortality rates in patients with serum ferritin levels below 200, between 200 and 500, and above 500 ng/mL were 23%, 9%, and 58%, respectively. The sensitivity and specificity for predicting mortality at a cut-off of 500 ng/mL were 64% and 90%, respectively.

In our study, similar to serum ferritin levels, the pSOFA scores were significantly higher among non-survivors compared to survivors (P < 0.05). ROC curve analysis showed the pSOFA score to be a significant predictor of mortality in children with sepsis (AUC: 0.9-1.0; p < 0.05). Both sensitivity and specificity of the mean pSOFA score at a cut-off of 12 were 100%. Thus, the diagnostic accuracy of serum ferritin was comparable to the pSOFA score in predicting mortality.

In a study by Baloch et al. [13], the cut-off value of the pSOFA score for predicting 30-day mortality was >2, with a sensitivity of 93.87%, specificity of 38.21%, and accuracy of 69.93%. Our study findings were also supported by the study by Aulia et al. [14], in which 93.3% of sepsis cases had a pSOFA score greater than 8, whereas 79.5% of non-sepsis cases had a pSOFA score less than 8 (P < 0.05). At the cut-off of 8 for predicting sepsis, AUC was 0.93, with 93.3% sensitivity and 79.5% specificity. Similarly, in the study by Mainling et al. [15], pSOFA scores were significantly higher among the non-survivor group (median 7.5; IQR: 6-11) compared to the survivor group (median 3; IQR: 2-4) (P < 0.05). A cut-off of 5 for the pSOFA score was found to be an excellent predictor of mortality (AUC: 0.937; 85.7% sensitivity, 87.7% specificity).

We also assessed the correlation between pSOFA scores and serum ferritin levels, and ROC curve analysis was conducted to determine hyperferritinemia using the pSOFA score. The mean pSOFA score showed a strong positive and significant correlation with serum ferritin levels (r = 0.70-0.90; p < 0.05). At a cut-off of 12, the pSOFA score was an excellent predictor of hyperferritinemia (>504 ng/mL) (AUC: 0.901; 79.5% sensitivity, 100% specificity). Our findings were supported by the study by Rusu et al. [16], in which a positive and significant correlation was found between serum ferritin levels and pSOFA scores (r = 0.7; 95% CI: 0.64-0.76; p < 0.01).

Our study precisely focused on a single specific biomarker, serum ferritin, which is a well-established acute-phase reactant. The presence of hyperferritinemia in children with severe sepsis may assist clinicians in early identification and timely intervention, thereby improving outcomes. Furthermore, our study evaluated the clinical correlation between serum ferritin levels and the pSOFA score, which further enhanced the prognostic value.

There are certain limitations to our study. It was conducted at a single center with a small sample size, limiting generalizability to the broader population. Additionally, serum iron studies could not be performed due to resource-limited settings.

## Conclusions

The study demonstrates that higher serum ferritin levels (>504 ng/mL) are a significant and independent predictor of mortality in children with severe sepsis. A pSOFA score >12 was associated with adverse outcomes in terms of mortality and was also found to be an independent predictor of hyperferritinemia (>504 ng/mL). As shown in the study, elevated ferritin levels can serve as a useful prognostic biomarker for severe sepsis. Further multi-center studies with larger sample sizes are required to better assess and validate the cutoff value for serum ferritin levels.
